# A Rare Case of Extra Gigantic Digit of Sole

**Published:** 2017-05

**Authors:** Chetan Satish, Subhash Reddy

**Affiliations:** Sanjeevani Hospital, Bangalore, India

**Keywords:** Gigantic digit, Sole, India

**DEAR EDITOR**

The classification of ulnar polydactyly exists of either two or three types. The two-stage classification according to Temtamy and McKusick involves type A and B. In type A there is an extra little finger at the metacarpophalangeal Joint, or more proximal including the carpometacarpal joint. The little finger can be hypoplastic or fully developed. Type B varies from a nubbin to an extra, non-functional little finger part on a pedicle. According to the three-type classification, type I includes nubbins or floating little fingers, type II includes duplications at the MCPJ, and type III includes duplications of the entire ray.^1^

We report a rare case of extra gigantic digit of sole in a boy. He was initially seen at age of 3 months when the parents brought him to us with complaints of an extra growth on right foot which was growing in size along with general growth of the baby. The peculiar thumb like appearance though on the ulnar aspect of foot was striking. Parents were concerned about the growth of the digit and more importantly they were concerned about the future walking of the child.

The baby was checked for any other congenital anomalies by pediatric team and anomaly screening was negative. It was decided to operate on the child at about 6 months of age. This was decided so that the developmental milestone of walking which is usually between 9 months to 1 year is not affected.

Radiological imaging showed the extra digit to have a well formed metatarsal bone and rudimentary phalanges. Surgical excision of the extra digit was undertaken and filleting of skin flap of extra digit was used to close the defect. Bone was disarticulated from the bifurcation of metatarsal joint. At present the child has started to stand with support at age of 9 months and has begun to take some baby steps to the delight of the parents.

Much of the initial growth and patterning of limbs occurs during weeks 4-8. Limb buds appear at about 4 weeks and much of the basic structures of the limbs (bones and muscle groups) are established by 8 weeks. After 8 weeks, the limb elements then just increase in size. Disruption of growth and/or patterning can result in many possible defects. Polydactyly:  extra digits (disruption –usually upregulation– of Shh pathway).^2^^,^^3^ This rare case is reported for gigantic ulnar polydactyly for 2 reasons. The first is the gigantic size of the digit with peculiar thumb like appearance and second to highlight the importance of excision of the digit at right age before the child takes the first baby steps that can be added to the literature.

## CONFLICT OF INTEREST

The authors declare no conflict of interest.
